# The beneficial Effects of *Eriobotrya Japonica* Lindl. Leaves Extract on Body Weight, Glycemic Control, and Lipid Profile

**DOI:** 10.2174/0115733998369417250426161250

**Published:** 2025-05-13

**Authors:** Ahmad Alkofahi, Karem H. Alzoubi, Omar F. Khabour, Mohammad A. Alzubi, Mohammad S. Shawaqfeh, Rania Mahafdeh

**Affiliations:** 1 Department of Medicinal Chemistry and Pharmacognosy, Faculty of Pharmacy, Jordan University of Science and Technology, Irbid, 22110, Jordan;; 2 Department of Pharmacy Practice and Pharmacotherapeutics, College of Pharmacy, University of Sharjah, Sharjah, UAE;; 3 Department of Clinical Pharmacy, Faculty of Pharmacy, Jordan University of Science and Technology, Irbid, Jordan;; 4 Department of Medical Laboratory Sciences, Faculty of Applied Medical Sciences, Jordan University of Science and Technology, Irbid, Jordan;; 5 College of Pharmacy, King Saud Bin Abdulaziz University for Health Sciences, Riyadh, Saudi Arabia;; 6 Department of Clinical Pharmacy, Faculty of Pharmacy, Jadara University, Irbid, 21110, Jordan

**Keywords:** High-fat diet, obesity, dyslipidemia, *Eriobotrya japonica* leaf, ethanolic extract, loquat, Jordan

## Abstract

**Introduction:**

*Eriobotrya japonica* Lindl, also known as loquat, is a subtropical fruit tree widely used in traditional Chinese medicine for thousands of years to treat many diseases such as asthma and chronic cough. Traditional healers also use it for weight reduction. In our present study, we validated the impact of *E. japonica* leaf extract on body weight, glycemic control, and lipid profile.

**Methods:**

The anti-obesity, anti-diabetic, and antihyperlipidemic effects of *E. japonica* leaf extract were screened in high-fat diet-fed rats. After treatment with different *E. japonica* leaf extract concentrations, body weight, blood glucose levels, HbA1c, and lipid profile were determined.

**Results and Discussion:**

The high-fat diet group treated with Loquat leaf extract >= 200 mg/ml/kg showed significant weight loss and a beneficial lipid marker profile. However, the glycemic markers were not significantly different.

**Conclusion:**

The findings demonstrate that *E. japonica* leaf extract at doses of ≥200 mg/kg significantly reduces body weight and improves lipid markers in HFD-fed rats. However, glycemic markers such as blood glucose and HbA1c were not significantly affected. These results highlight the potential of *E. japonica* as a natural weight loss and lipid control aid, warranting further investigation into its mechanisms and clinical applicability.

## 1 INTRODUCTION

Natural products, such as plants, have been the fundamental sources of treatment for human diseases [1, 2]. Ancient wisdom has been an essential source of drug discovery, contributing significantly to modern medicine [3-5]. Approximately 60% of currently approved drugs for antitumor and anti-infective agents are derived from natural compounds [6]. Many natural compounds have shown promising effects in managing diabetes, obesity, and dyslipidemia [7]. *Eriobotrya japonica* Lindl. (loquat) has gained attention for its potential therapeutic properties [3, 8]. Loquat is an evergreen subtropical fruit mostly grown in southeastern China that is cultivated in over 30 countries worldwide [9], including Italy, Japan, Spain, Turkey, India, Brazil, and Pakistan [10-13] loquat fruit ripens from May through June, giving producers a long-term source of fresh fruit and a significant economic benefit [11, 14, 15]. Loquats have been utilized in traditional medicine for centuries due to their high medicinal value [16-18]. Loquat has been used as a potential treatment for diabetes, dyslipidemia [10, 14, 19, 20] different inflammatory diseases [11, 21, 22], antifungal [23, 24], antimicrobial [25, 26], and chronic bronchitis (CB) [27, 28]. The phytonutrient composition of organ extracts varies significantly across different plants [11, 29]. Loquat leaves and flowers are abundantly rich in triterpenes and phenolics [30]. The fruits are rich in flavonoids, organic acids, phenolic acids, carotenoids, and vitamins [11, 31]. While the kernel is rich in starch, proteins, tannins, and minerals [11, 32, 33].

Despite the availability of pharmacological treatments for obesity, many anti-obesity medications are associated with significant side effects, limited long-term efficacy, and high costs [34, 35]. These limitations have led to increased interest in natural alternatives, such as medicinal plants with bioactive compounds that may offer safer and more sustainable approaches to weight management. Loquats have antioxidant bioactive compounds, which may be considered a promising alternative therapy for diabetes, obesity, and dyslipidemia [11, 36, 37]. This study aims to investigate the influence of loquats (*Eriobotrya japonica*) leaf extract on body weight, glycemic control, and lipid profile. To our knowledge, no studies have determined the optimal dose of the loquats leaf extract to achieve a therapeutic effect for treating obesity, hyperglycemia, and hyperlipidemia. This study could be the basis for future use of loquats in controlling weight, glucose, and lipid.

## 2 MATERIALS AND METHODS

### 2.1 Collection and Identification of the Plant

The plant was collected from the home garden of one of the authors in Irbid City, north of Jordan, during the summer months between May and August. The collected plant was authenticated by Dr. Jameel Laaham, a plant taxonomist at the Department of Biological Sciences at the Faculty of Science, Yarmouk University, Irbid, Jordan. A voucher specimen of the plant was deposited at the Department of Medicinal Chemistry and Pharmacognosy at the Faculty of Pharmacy, Jordan University of Science and Technology, Irbid, Jordan.

### 2.2 Preparation of Plant Extract

The plant was dried in the shade and then ground using a Wiley grinder (mesh size is 2 mm diameter). A 95% ethanol solution was used to percolate the ground material several times until the entire ground substance was extracted, and the resulting ethanolic extract was concentrated under vacuum to yield the sticky extract [38, 39].

### 2.3 Animals and Housing Conditions

Wistar male rats weighing 200-250g (10-12 weeks) were purchased from the Animal House at Jordan University of Science and Technology, Irbid, Jordan. The animals were randomly divided into experimental groups, with each group consisting of 15 rats. The animals were kept in metal cages (size of the cages: LxWxH (cm): 55x45x25) and kept under a hygienic environment at 25 ± 1°C with unrestricted access to drinking water and a standard diet. Animals' tails have been labeled using a waterproof marker and maintained at a 12:12 hours, dark-light cycle. The animals were housed in cages for two weeks to ensure social acclimatization before starting the experiments. The animal experiments were carried out according to the Animal Care and Use Committee (ACUC) protocol at the Jordan University of Science and Technology, Irbid, Jordan. This study adheres to internationally accepted standards for animal research, following the 3Rs principle. The ARRIVE guidelines were employed to report experiments involving live animals and promote ethical research practices.

### 2.4 Animal Groups and Treatment

Rats were randomly divided into five groups (n= 15 rats for each group) [1]: the control group (n=15 rats), was treated with a vehicle and fed a standard diet [2]; the HDF group (n=15 rats), was administered a high-fat diet (HFD)/ Groups 3, 4, and 5 were the treated groups, fed an HFD and simultaneously administered the plant extract *via* oral gavage at doses of 50 mg/ml/kg (Group 3), 200 mg/ml/kg (Group 4), and 500 mg/ml/kg (Group 5). The treatment continued for 16 weeks. HFD contained (g%): total fat (25%), unsaturated fats (11%), carbohydrates (44%), protein (18%), fibers (13%), and other ingredients. The different groups were fed a standard diet containing (g%): total fat (5%), unsaturated fats (2%,) carbohydrates (62%), proteins (20%), fibers (13%), and other ingredients [Alzoubi, 2018 #^10^]. Two diets contain identical amounts of essential vitamins, minerals, and nutrients. Throughout the trials, food was available for the duration of the experiments.

### 2.5 Body Weight, Blood Glucose, and Lipid Profile

Throughout the experimental time frame, the rats were weighed every week. When the experiment finished, rats were sacrificed without anesthesia since anesthesia affects the lipid profile, so blood was collected in EDTA tubes. Plasma samples were obtained from the blood samples by centrifuging and kept at −30°C until analysis. The total cholesterol (TC), Low-Density Lipoprotein (LDL), High-Density Lipoprotein (HDL), Triglycerides (TG), blood glucose, and HbA1c were measured using Roche Analyzer & Roche reagents (Roche Diagnostics, Basel, Switzerland) {Alomari, 2020 #11}.

### 2.6 Statistical Analysis

The statistical analyses and graphs were done using GraphPad Prism version 4 (GraphPad Software, LA Jolla, CA). Two-way ANOVA was used to analyze body weight data, whereas one-way ANOVA followed by Tukey’s post-test was used for all other comparisons. A *p* < 0.05 was set to be significant in all statistics. All results were presented as mean ± SEM.

## 3 RESULTS

### 3.1 *E. japonica* Leaf Extract's Impact on Body Weight

All groups except the control group were administered HFD. The treated group was fed the HFD and simultaneously treated with different doses of *E. japonica* extracts: 50, 200, and 500 mg/ml/kg *via* oral gavage. The control group was treated with a vehicle and fed a standard diet. Treatment continued for 16 weeks. The rats' body weight was measured weekly. (Fig. **1**) shows significant differences in body weight starting from week 6 in the group treated with 200 and 500 mg/ml/kg of *E. japonica* leaf extract compared to the HFD and the HFD-E50 groups (*p* < 0.05). The body weight of HFD-fed groups increased starting from week 6 and onwards. However, the rats treated with 200 and 500 mg/ml/kg of *E. japonica* leaf extract (HFD-E200, HFD-E500) showed a significant decrease in body weight compared to the HFD groups that were not treated with the extract. This trend continued through the end of the treatment period in week 16. Interestingly, the average body weights of the HFD-E200 and HFD-E500 groups were close to the control groups, suggesting that the *E. japonica* leaf extract effectively mitigated the weight gain typically associated with a high-fat diet. No significant differences were observed in daily food or water consumption across all groups, indicating that the observed effects on body weight were not due to differences in dietary intake.

### 3.2 *E. japonica* Leaf Extract's Impact on Organ Weight

The impact of *E. japonica* leaf extract on organ weight was assessed by measuring the weights of various organs in all experimental groups. The organs measured included the liver, heart, lungs, kidneys, spleen, and brain. Following a 16-weeks of treatment, these measures were made. The results are illustrated in Fig. (**2**). There is no significant difference in all results except for the liver weight in the HFD group, which received no extract, which was significantly greater than the control group. The groups were fed HFD and treated with 200 and 500 mg/ml/kg of leaf extract (*p* 0.05). This indicates that the administration of *E. japonica* leaf extract provided a protective effect against the liver weight increase typically induced by HFD. There are no significant differences in the liver weights between the control group and the HFD groups treated with 200 and 500 mg/ml/kg of the extract, suggesting that the extract mitigated the liver weight gain associated with the high-fat diet.

### 3.3 *E. japonica* Leaf Extract's Impact on Glycemic and Lipid Markers

The impact of *E. japonica* leaf extract on glycemic and lipid markers was assessed by measuring various biomarkers in all experimental groups and comparing them to the control group. The markers measured included blood glucose, HbA1c, TC, TG, LDL, and HDL levels. As shown in Fig. (**3**), the blood glucose and HbA1c levels did not exhibit significance between the groups, indicating that *E. japonica* leaf extract did not affect these glycemic markers. However, significance was seen in the lipid markers. TC and TG levels were significantly elevated in the HFD groups and HFD groups receiving 50 mg/ml/kg (HFD+E50) than in the control group. Similarly, LDL levels were significantly elevated among the HFD and HFD+E50 groups. In contrast, the HDL levels were significantly lower among the HFD and HFD+E50 groups than in the control group. These findings indicate that while the 50 mg/ml/kg of *E. japonica* leaf extract does not significantly impact glycemic markers, it does not mitigate the adverse effects of HFD on lipid markers. Higher doses may be necessary to observe a protective effect on these lipid parameters.

## 4 DISCUSSION

In this study, we examined the impact of *E. japonica* leaf extract on body weight, glycemic control, and lipid profile in an HFD-induced obesity model. However, the glycemic markers did not show significant benefits; our findings highlighted the potential therapeutic benefits of *E. japonica* leaf extract at doses of 200 mg/ml/kg and above in managing obesity.

In present experiments, daily consumption of *E. japonica* leaf extract (at doses = 200 mg/ml/kg) improved the lipid profile, specifically lowering TC, LDL, and TG while increasing HDL levels in serum, suggesting that *E. japonica* leaf extract reduces dyslipidemia induced by HFD. Additionally, *E. japonica* leaf extract at doses of 200 mg/ml/kg and above reduces body and liver weight, suggesting that *E. japonica* leaf extract can reduce central obesity induced by HFD. Our results are aligned with previous studies. Recently, Abdelrahman *et al.*, (2023) [40] demonstrated that *E. japonica* leaf ethanolic extract of 100 and 400 mg/kg, notably lowered the body weight and effectively prevented hyperlipidemia in HFD-fed rats over 12 weeks. Similarly, Li *et al.* (2020) [41] showed that administering 200 mg/ml/kg of *E. japonica* leaf extract *via* oral gavage to C57BL/6 J mice given HFD for 12 weeks can successfully reduce liver weight and visceral central obesity, insulin resistance, and dyslipidemia. Furthermore, *E. japonica* leaf extracts not only decreased the weight but also effectively inhibited lipid accumulation, significantly reducing the body weight and TG levels compared to those of the control group [42]. Additionally, Mokhtari *et al.* 2023, demonstrated that *E. japonica* fruit extract could ameliorate hyperlipidemia and related toxic tissue complications by significantly preventing oxidative stress and restoring lipid metabolism [43] Kim *et al.* (2012) noted that *E. japonica* extract had significant anti-diabetic activity and anti-atherosclerotic *in vitro* and *in vivo* in hyperlipidemic zebrafish models [44]. However, the previous researcher used different *in vivo* models, extracts, and doses of, *E. japonica*; it is still consistent with our findings in anti-obesity and antihyperlipidemic drugs.

Different mechanisms are involved in exhibiting the antihyperlipidemic and weight regulation effects of *E. japonica* [45, 46]. Flavonoids and triterpenes, the major bioactive compounds in *E. japonica*, have been demonstrated to control lipid metabolism and energy homeostasis [36, 47-49]. Flavonoids may exert anti-obesity effects by modulating AMP-activated protein kinase (AMPK) signaling [50, 51], which plays a crucial role in lipid oxidation and glucose metabolism [45, 52] Additionally, they may inhibit key enzymes involved in lipid synthesis [53, 54], such as acetyl- CoA carboxylase (ACC) [55] and fatty acid synthase (FAS) [56], reducing lipid accumulation [57-59].

Another of these mechanisms is to control obesity by regulating appetite through neurological and hormonal mechanisms [40, 60] Previous studies suggest that certain bioactive compounds in *E. japonica* may influence leptin and ghrelin levels, which are key regulators of hunger and satiety [45, 61, 62] However, in our study, food and water consumption remained unchanged among all groups during the experimental period. This indicates that alternative metabolic pathways may be responsible for the observed reductions in weight and lipid levels.

Moreover, the anti-inflammatory and antioxidant effects of *E. japonica* may have a crucial role in lowering lipid profiles [3, 63, 64]. *E. japonica* has demonstrated the ability to reduce pro-inflammatory cytokines such as TNF-α and IL-6, thereby improving metabolic health [28, 65, 66] An additional mechanism of lowering metabolic syndrome signs is through preventing glucose absorption [3, 30, 67]. For example, Song *et al.*, (2019) [68] presented that flavonoids can inhibit digestive catalysts such as α-glucosidase, thereby preventing the absorption of excess glucose, which contributes to controlling blood glucose levels and offers an additional approach to preventing obesity and diabetes. However, our study observed significant benefits in lipid markers but not in glycemic markers with *E. japonica* leaf extract. Accumulated evidence has documented the positive effects of *E. japonica* on glycemic markers such as insulin resistance and HbA1c [58, 69-71] however, our findings contrast with this aspect. Specifically, no significant changes were observed in blood glucose or HbA1c levels across all treatment groups over the 16 weeks, suggesting that the concentration of the *E. japonica* leaf extract was insufficient to influence these markers. The variance in our findings compared to the existing literature may be attributed to differences in *in-vivo* models and extract doses utilized. For instance, Abdelrahman *et al.*, (2023) noted that the ethanolic extract of *E. japonica* leaves had no significant effect on glucose levels in rats fed HFD at 40, 100, or 400 mg/kg. In contrast, a significant decrease in blood glucose levels was shown with the fruit extract at the same doses, indicating that loquat fruits may yield more potent extracts than leaves or seeds because of higher concentrations of bioactive compounds. Notably, other studies presenting the antidiabetic efficacy of loquat leaves have focused on sesquiterpene-rich extracts [72-76] For example, extracts rich with sesquiterpene glycoside from loquat have been shown to have a hypoglycemic effect on diabetic rats [73]. While our study did not demonstrate significant antidiabetic effects with the ethanolic extract of loquat leaves, the differences in observed outcomes across studies highlight the importance of the specific bioactive components, extract concentrations, and experimental models used. This underscores the need for further follow-up studies to develop an effective diabetes model, optimize these parameters, and fully elucidate the potential antidiabetic benefits of *E. japonica*.

This animal study sheds light on the probable utilization of loquat, a commonly available plant in Jordan, for weight reduction. The promising results from the loquat extract highlight the significance of our research, suggesting its potential in weight reduction, hyperlipidemia, and diabetes management. These findings open the door for further studies to exhibit the exact mechanism behind the dose-dependent anti-diabetic, ant-hyperlipidemic, and ant-obesity effects of loquat extract.

## CONCLUSION

Our findings suggest that *E. japonica* leaf extract effectively ameliorates HFD-induced hyperlipidemia and central obesity in Wistar rats. The extract demonstrated a significant impact on lipid regulation and weight management due to its bioactive compounds. These results highlight the potential of *E. japonica* as a natural therapeutic agent for metabolic disorders, including obesity and dyslipidemia.

However, further studies are required to elucidate the exact mechanisms underlying its effects, particularly at the molecular and histological levels. Additionally, GC-MS/LC-MS analysis is necessary to identify the specific bioactive compounds responsible for the observed benefits. Future research should also explore its efficacy in clinical settings to determine its translational potential for human health.

## LIMITATIONS OF THE STUDY

Our study provides valuable insights into the potential anti-obesity and antihyperlipidemic effects of *E. japonica* leaf extract. However, several limitations must be mentioned. First, although we observed significant improvements in lipid profiles and body weight, we did not conduct histological examinations of key organs such as the liver, adipose tissue, or pancreas to assess structural or cellular changes. Future studies should include histopathological analyses to better understand the mechanisms involved. Third, the extract's phytochemical composition was not fully characterized using GC-MS or LC-MS analysis, limiting our ability to link specific bioactive compounds to the observed effects. Identifying the key active compounds is essential for further pharmacological validation. Finally, while we explored metabolic markers, we did not investigate molecular mechanisms such as gene expression, enzymatic activity, or signaling pathways involved in lipid metabolism and weight regulation. Future research should focus on uncovering the underlying molecular mechanisms to provide a more comprehensive understanding of *E. japonica's* therapeutic potential.

## Figures and Tables

**Fig. (1):**
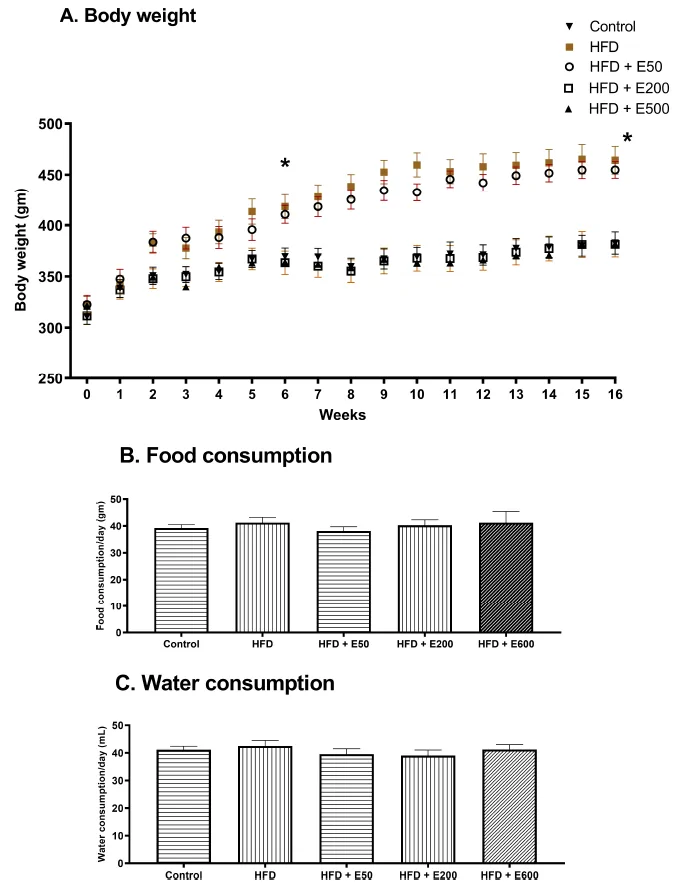
Body weight changes over 16 weeks with oral administration of *E. japonica* leaf extract (**A**). Groups: control (standard diet), HFD, and HFD with extract (HFD+E50, HFD+E200, and HFD+E500 mg/kg). Statistical analysis was performed using GraphPad Prism version 4. Body weight data was analyzed using two-way ANOVA, while one-way ANOVA followed by Tukey’s post-hoc test was used for all other comparisons. Significant weight differences were observed in the 200 and 500 mg/kg groups from week 6 compared to the HFD and 50 mg/kg groups (*p* < 0.05). Asterisks (*) indicate statistically significant differences between groups. No significant differences were found in daily food or water intake among the groups (**B** and **C**) (*p* > 0.05). Data is presented as mean ± SEM (n = 15 per group).

**Fig. (2):**
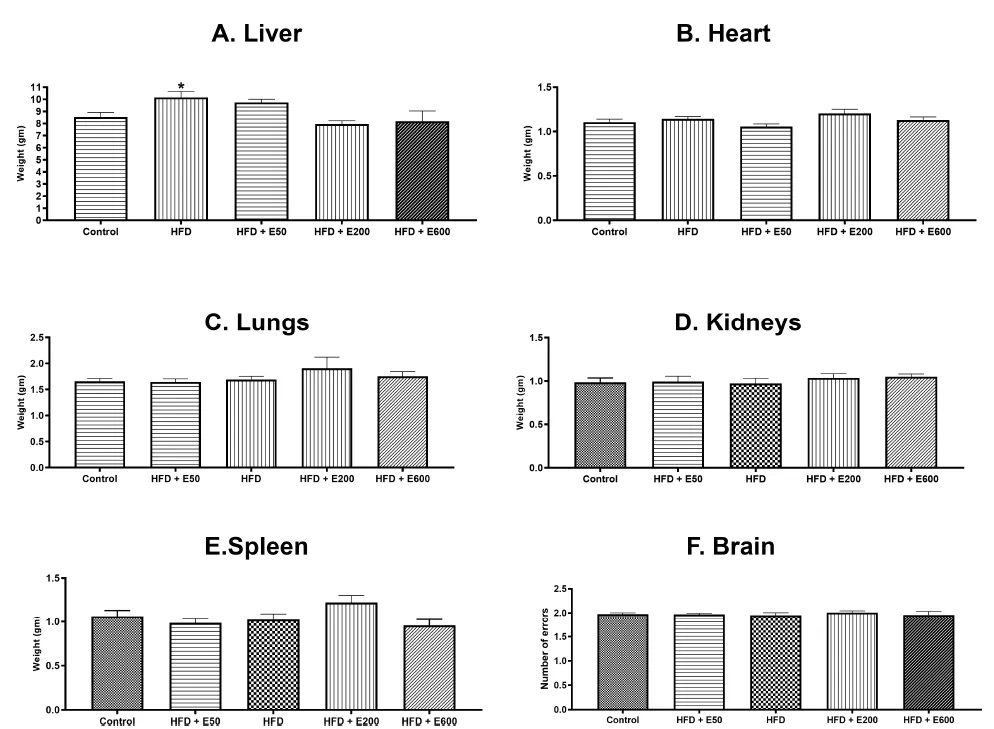
Organ weight measurements after 16 weeks of oral E. japonica leaf extract administration. Groups: control (standard diet), HFD, and HFD with extract (HFD+E50, HFD+E200, and HFD+E500 mg/ml/kg). Statistical analysis was performed using GraphPad Prism version 4. One-way ANOVA followed by Tukey’s post-hoc test was used for all comparisons. (**A**) Liver weight showed significant differences in the HFD group compared to the control, HFD+E200, and HFD+E500 mg/kg groups, indicating a protective effect of the extract against liver weight increase due to the high-fat diet (p 0.05). (**B-F**) No significant differences were observed among the weights of the heart, lungs, kidneys, spleen, and brain across all groups (p > 0.05). Data is presented as mean ± SEM (n = 15 per group). Asterisks (*) indicate statistically significant differences between groups.

**Fig. (3):**
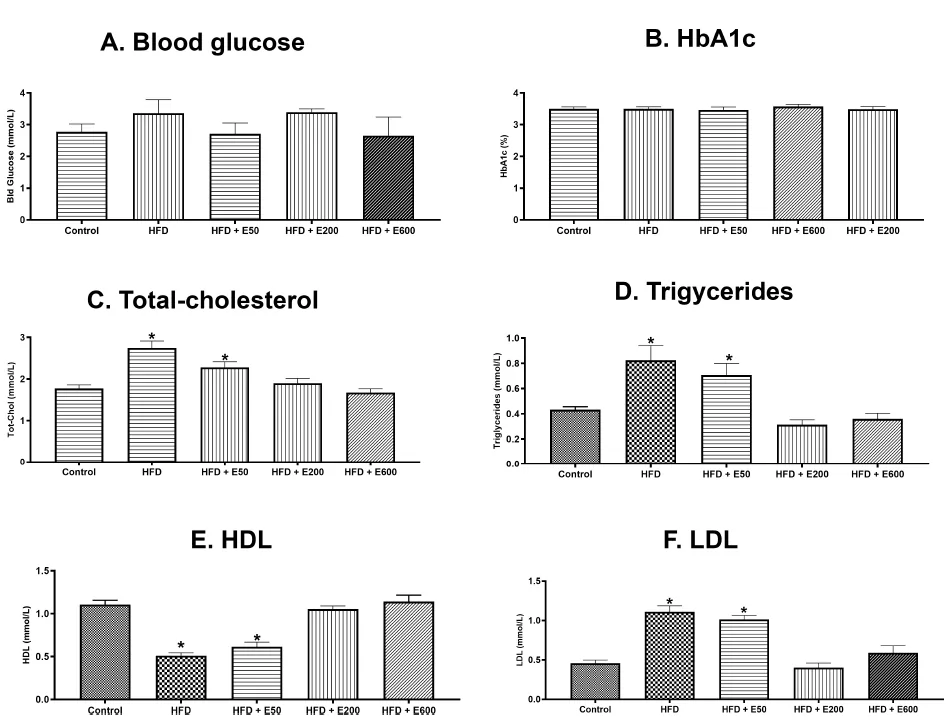
*E. japonica* leaf extract's impact on glycemic and lipid markers after 16 weeks. Groups: control (standard diet), HFD, and HFD with extract (HFD+E50, HFD+E200, and HFD+E500 mg/ml/kg). Statistical analysis was performed using GraphPad Prism version 4. One-way ANOVA followed by Tukey’s post-hoc test was used for all comparisons. (**A**) Blood glucose and (**B**) HbA1c levels showed no significant differences across all groups (*p* 0.05). (**C** **D**) Total cholesterol (TC) and triglyceride (TG) levels were significantly elevated in the HFD and HFD+E50 groups compared to the control group (*p* 0.05). (**E**) LDL levels were significantly higher in the HFD and HFD+E50 groups (*p* 0.05). (**F**) HDL levels were significantly lower in both the HFD and HFD+E50 groups compared to the control (*p* 0.05). Data is presented as mean ± SEM (n = 15 per group). * Shows a significant difference between the groups (*p* 0.05).

## Data Availability

All data generated or analyzed during this study are included in this published article.

## LIST OF ABBREVIATIONS

CB: Chronic Bronchitis
HFD: High-fat Diet
TC: Total Cholesterol
LDL: Low-Density Lipoprotein
ACC: Acetyl-CoA Carboxylase
AMPK: AMP-activated Protein Kinase
TG: Triglycerides
HDL: High-Density Lipoprotein
